# LncRNA GASL1 is downregulated in chronic heart failure and regulates cardiomyocyte apoptosis

**DOI:** 10.1186/s11658-019-0165-x

**Published:** 2019-06-13

**Authors:** Haihong Deng, Wenbo Ouyang, Li Zhang, Xiaoshan Xiao, Zhiyong Huang, Wendian Zhu

**Affiliations:** 1 Department of Anesthesiology, The First People’s Hospital of Zhaoqing, Zhaoqing City, Guangdong Province 526000 People’s Republic of China; 2 Department of General Surgery, The First People’s Hospital of Zhaoqing, No. 9 Donggang East Road, Duanzhou District, Zhaoqing City, Guangdong Province 526000 People’s Republic of China; 3 Department of Anesthesiology, Fuwai Hospital Chinese Academy of Medical Sciences, No. 12 Langshan Road, Shenzhen City, 518057 People’s Republic of China; 4 Department of Anesthesiology, Guangdong No. 2 Provincial People’s Hospital, Guangdong Provincial Emergency Hospital, Guangzhou City, Guangdong Province 510317 People’s Republic of China

**Keywords:** Chronic heart failure, lncRNA GASL1, TGF-β1, Apoptosis

## Abstract

**Background:**

TGF-β1 contributes to chronic heart failure. It is known that lncRNA GASL1 can inactivate TGF-β1 in cancer biology.

**Methods:**

All the participants were enrolled in the First People’s Hospital of Zhaoqing during the period June 2012 to June 2013. ELISA, RT-qPCR, vectors, transient transfections and western blot were carried out during the research.

**Results:**

We found that plasma levels of TGF-β1 were significantly higher, while levels of GASL1 in plasma were significantly lower in chronic heart failure (CHF) patients compared to the control group. TGF-β1 and GASL1 were inversely correlated in CHF patients. Low pretreatment plasma levels of GASL1 were closely associated with poor survival of CHF patients. GASL1 expression was not significantly affected by TGF-β1 overexpression in cardiomyocytes, while cardiomyocytes with GASL1 overexpression showed downregulated TGF-β1. Overexpression of GASL1 led to a decreased, while TGF-β1 overexpression led to an increased apoptotic rate of cardiomyocytes under H_2_O_2_ treatment. In addition, TGF-β1 overexpression attenuated the effect of GASL1 overexpression.

**Conclusion:**

In conclusion, GASL1 was downregulated in CHF. GASL1 overexpression may improve CHF by inhibiting cardiomyocyte apoptosis through the inactivation of TGF-β1.

## Background

Heart diseases cause more deaths than the sum of all types of cancer [1]. In effect, heart diseases, such as chronic heart failure (CHF), are the leading cause of hospital admission in many regions of the world [2]. In the United States, CHF is responsible for 1 out of 9 deaths [3], and 35 billion US dollars are spent on its prevention and treatment [4]. Occurrence of CHF is closely correlated with many other clinical disorders, such as hypercholesterolemia, hypertension, and diabetes mellitus [5]. With the growth of aging population, the incidence rate of CHF is predicted to further increase all over the world [5]. Therefore, development of novel therapeutic targets is urgently needed to improve the survival of CHF patients.

Studies on heart failures have revealed that many factors are related to the disease development, while genetic factors play central roles in this process [6, 7]. Long non-coding RNAs (lncRNAs, > 200 nt) have critical roles in heart failure by regulating expression of related genes [8]. GASL1 is a recently characterized tumor suppressive lncRNA in cancer biology [9, 10]. A recent study reported that GASL1 regulated lung cancer cell growth by inactivating TGF-β1 [10], which contributes to the development of heart failure [11]. We therefore investigated the roles of GASL1 in CHF.

## Materials and methods

### Patients

The patient group in this study included 72 CHF patients (40 males and 32 females, 44 to 74 years, 56.6 ± 6.3 years). The control group included 66 healthy volunteers (40 males and 32 females, 44 to 74 years, 56.6 ± 6.3 years). All those participants were enrolled in the First People’s Hospital of Zhaoqing during the period June 2012 to June 2013. Patients complicated with other clinical disorders, with history of malignancies, who received any therapies within 100 days before treatment were excluded from this study. The age and gender distributions were not significantly different between patient and control groups. The Ethics Committee of the First People’s Hospital of Zhaoqing approved this study before the admission of patients and controls. All participants signed informed consent.

### Plasma and cell lines

Fasting blood (5 ml) was collected from each patient and control before the initiation of therapies. Blood samples were injected into EDTA tubes, and the tubes were centrifuged at 1200 g for 15 min to collect plasma.

AC16 human cardiomyocyte cell line (EMD Millipore, USA) was used. DMEM containing 1% penicillin and streptomycin as well as 12% fetal bovine serum (FBS) was used as cell culture medium. Cell culture conditions were 37 °C and 5% CO_2_.

### Follow-up

A 5-year follow-up study was carried out to monitor the survival of all 72 CHF patients. Follow-up was carried out mainly by telephone, and an outpatient visit was performed in some cases. Patients who died of other causes, such as other diseases or traffic accidents, were excluded from this study.

### Elisa

TGF-β1 in plasma was detected by performing ELISA experiments using Human TGF-β1 Quantikine ELISA Kit (DB100B, R&D Systems). Sensitivity of this kit was 15.4 pg/ml. Levels of TGF-β1 in plasma were normalized to ng/ml.

### RT-qPCR

Total RNA extractions from plasma and AC16 cells were performed using Ribozol (Thermo Fisher Scientific) reagent. Synthesis of cDNA was performed through reverse transcriptions using the RevertAid RT Reverse Transcription Kit (Thermo Fisher Scientific). All qPCR mixtures were prepared with the SYBR Green Quantitative RT-qPCR Kit (Sigma-Aldrich). 18 s rRNA or GAPDH was used as an endogenous control to normalize GASL1 and TGF-β1 expression. All PCR reactions were repeated 3 times. Data were processed using the 2^-ΔΔCT^ method.

### Vectors and transient transfections

GASL1 and TGF-β1 overexpression vectors (pcDNA3.1) were constructed by Sangon (Shanghai, China). AC16 cells were cultivated to confluence of 70–80% and transient cell transfections were performed using Lipofectamine 2000 reagent (Thermo Fisher Scientific) with 10 nM vector. Cells without transfections (control) and empty vector-transfected cells (negative control) were included as two controls.

### Measurement of apoptotic assay

AC16 cells were harvested at 24 h after transfections. Cells were mixed with DMEM to prepare single cell suspensions (3 × 10^4^ cells/ml). A 6-well plate was used to cultivate the cells with 2 ml of cell suspension in each well. 150 μM H_2_O_2_ was then added into each well. Cells were cultivated for 24 h, followed by digestion with 0.25% trypsin. Finally, staining with propidium iodide (PI) and Annexin V-FITC (Dojindo, Japan) was performed and apoptotic cells were detected by performing flow cytometry.

### Western blot

AC16 cells were harvested at 24 h after transfections and total protein was extracted using RIPA solution (Sangon, Shanghai, China). Protein samples were denatured and 10% SDS-PAGE gel electrophoresis was performed. Following gel-transfer to PVDF membrane, blocking was performed in 5% fat-free milk for 2 h at room temperature. After that, membranes were incubated with TGF-β1 (1: 1300, ab92486, Abcam) and GAPDH (1: 1300, ab8245, Abcam) primary antibodies at 4 °C overnight. Following that, membranes were further incubated with IgG-HRP goat anti-rabbit secondary antibody (1: 900, MBS435036, MyBioSource) for 2 h at room temperature. Signals were developed using ECL (Sigma-Aldrich) and data were normalized using Image J v1.46 software.

### Statistical process

To obtain solid data, all experiments were repeated 3 times. Differences between patient and control groups were performed by performing an unpaired t test. Differences among different cell transfection groups were analyzed by ANOVA (one-way) and Tukey test. Correlations between GASL1 and TGF-β1 were analyzed by linear regression. Based on plasma levels of GASL1, the 72 CHF patients were grouped into high (*n* = 35) and low (*n* = 37) groups (Youden’s index). K-M plotter was used to draw survival curves, which were compared using the log-rank test. The level of statistical significance was *p* < 0.05.

## Results

### Altered levels of TGF-β1 and GASL1 were observed in CHF patients

**Fig. 1:**
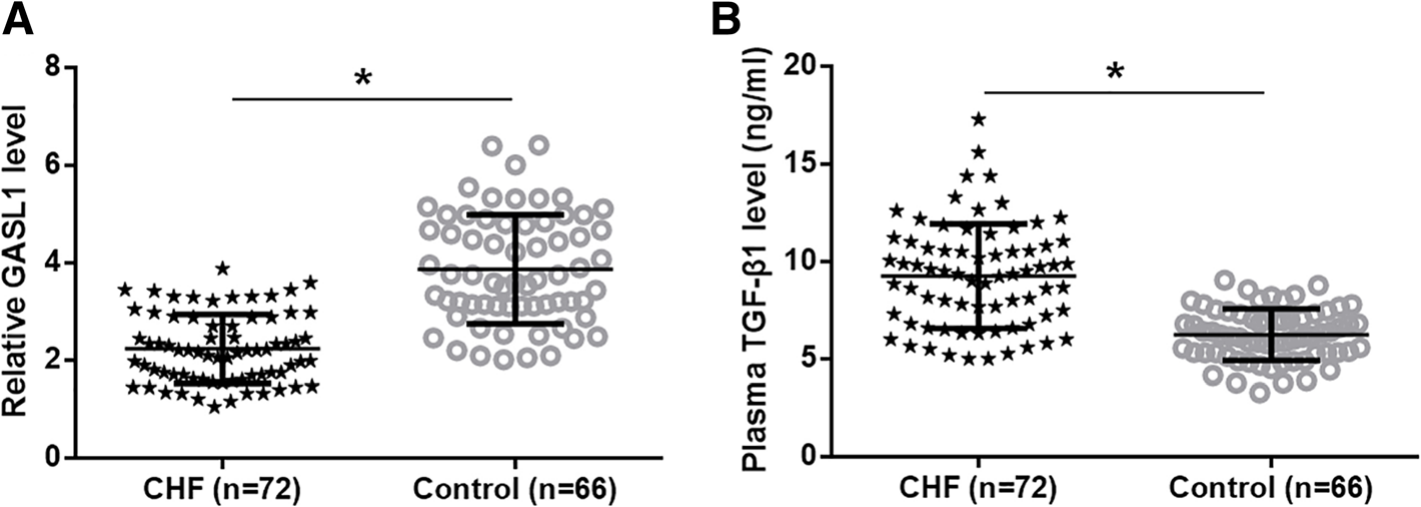
Altered levels of TGF-β1 and GASL1 were observed in CHF patients. Analysis of ELISA and RT-qPCR data by unpaired t test showed that levels of TGF-β1 in plasma were significantly higher (**a**), while plasma levels of GASL1 were significantly lower (**b**) in CHF patients than in healthy controls (*, *p* < 0.05)

TGF-β1 and GASL1 in plasma were detected by ELISA and RT-qPCR experiments, respectively. Differences of plasma levels of TGF-β1 and GASL1 were analyzed by performing an unpaired t test. It was found that plasma levels of TGF-β1 were significantly higher (Fig. 1a, *p* < 0.05), while plasma levels of GASL1 were significantly lower (Fig. 1b, *p* < 0.05) in CHF patients than in healthy controls.

### TGF-β1 and GASL1 were inversely correlated

**Fig. 2:**
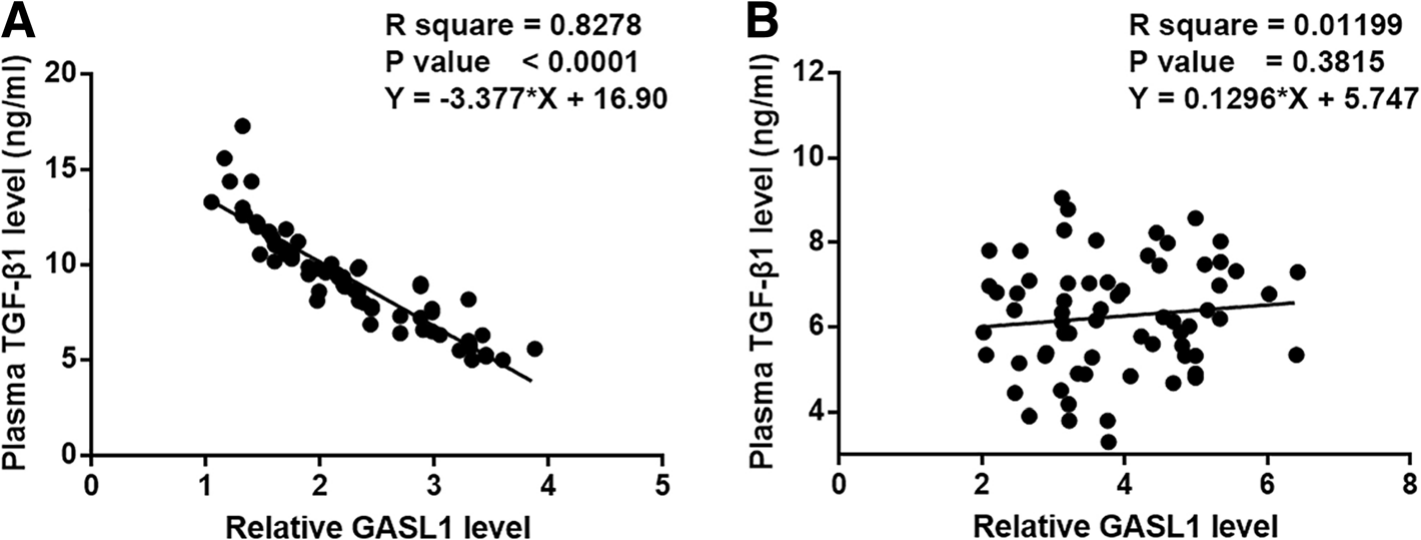
TGF-β1 and GASL1 were inversely correlated. Levels of TGF-β1 and GASL1 in plasma were inversely correlated in CHF patients (**a**), but not in control group (**b**)

Correlations between GASL1 and TGF-β1 were analyzed by linear regression. Plasma levels of TGF-β1 and GASL1 were found to the significant and inversely correlated in CHF patients (Fig. 2a). However, in healthy controls, TGF-β1 and GASL1 were not significantly correlated (Fig. 2b).

### Low plasma levels of GASL1 were closely correlated with poor survival

**Fig. 3:**
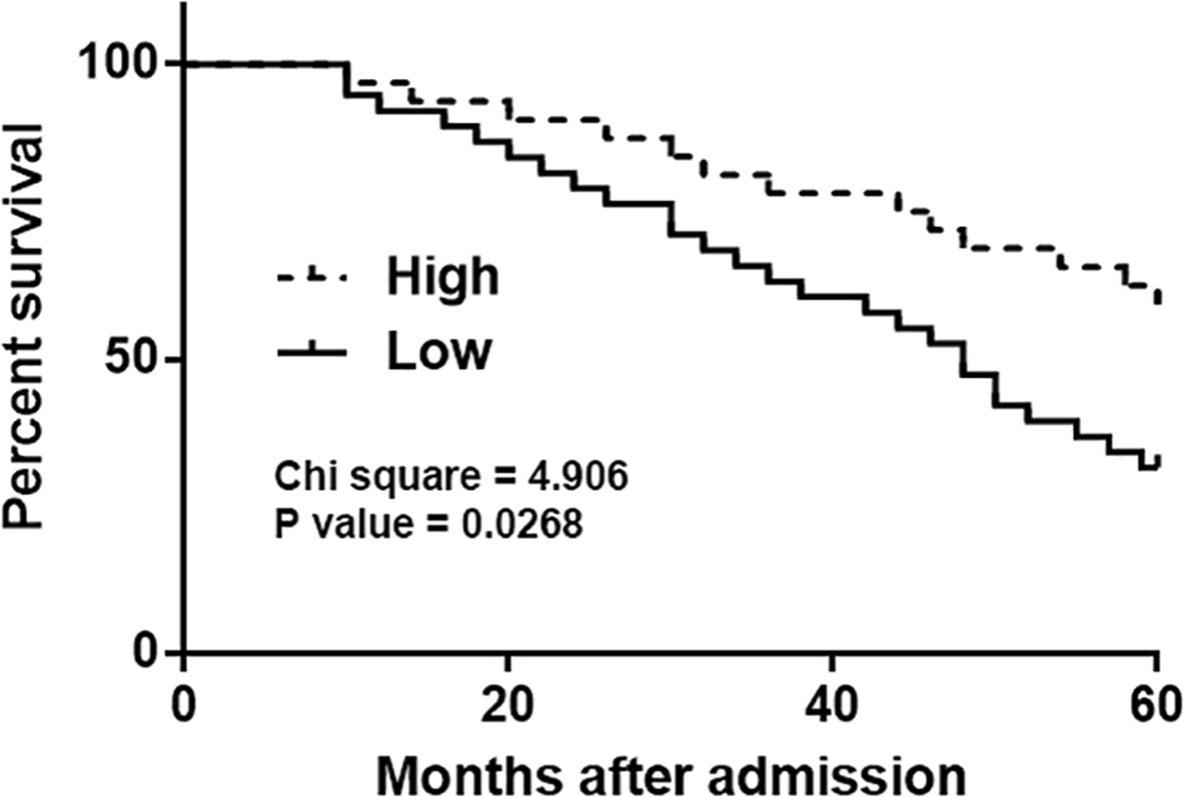
Low plasma levels of GASL1 were closely correlated with poor survival. Analysis of survival data showed that low plasma levels of GASL1 were closely correlated with poor survival

Based on plasma levels of GASL1, the 72 CHF patients were grouped into high (*n* = 35) and low (*n* = 37) groups (Youden’s index). The K-M method and log-rank test were used to plot and compare the survival curves. It was found that patients with low plasma levels of GASL1 had a significantly lower overall survival rate compared to patients with a high plasma GASL1 level (Fig. 3).

### GASL1 downregulated TGF-β1 to inhibit AC16 cell apoptosis

**Fig. 4:**
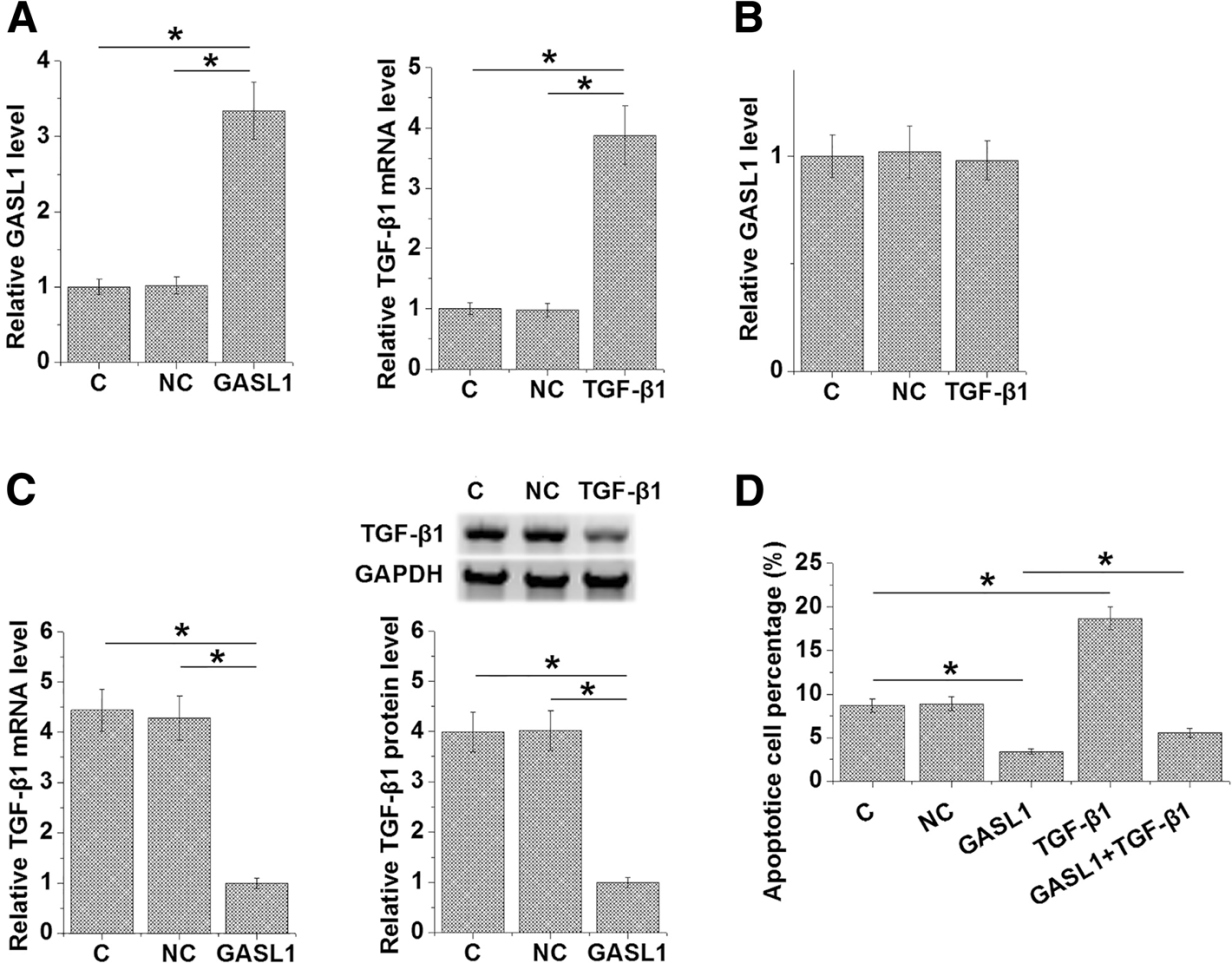
GASL1 downregulated TGF-β1 to inhibit AC16 cell apoptosis. Expression data analysis showed that expression levels of TGF-β1 and GASL1 were significantly increased in AC16 cells compared to two controls (Control, C; Negative control, NC) at 24 h after transfections (**a**). In addition, TGF-β1 overexpression failed to affect GASL1 in AC16 cells (**b**), while GASL1 overexpression mediated the downregulation of TGF-β1 at both mRNA and protein levels (**c**). Cell apoptotic data analyzed by one-way ANOVA and Tukey test showed that overexpression of GASL1 led to decreased, while TGF-β1 overexpression led to increased apoptotic rate of cardiomyocytes under H_2_O_2_ treatment. In addition, TGF-β1 overexpression attenuated the effect of GASL1 overexpression (**d**) (*, *p* < 0.05)

At 24 h after transfections, expression data were analyzed by one-way ANOVA and Tukey test to find the differences among groups. Expression levels of TGF-β1 and GASL1 were significantly increased in AC16 cells compared to two controls (Control, C; Negative control, NC; Fig. 4a; *p* < 0.05). In addition, TGF-β1 overexpression failed to affect GASL1 in AC16 cells (Fig. 4b), while GASL1 overexpression mediated the downregulation of TGF-β1 at both mRNA and protein levels (Fig. 4c, *p* < 0.05). Cell apoptotic data analyzed by one-way ANOVA and Tukey test showed that overexpression of GASL1 led to a decreased, while TGF-β1 overexpression led to an increased apoptotic rate of cardiomyocytes under H_2_O_2_ treatment. In addition, TGF-β1 overexpression attenuated the effect of GASL1 overexpression (Fig. 4d, *p* < 0.05).

## Discussion

In this study, we investigated the role of GASL1 in CHF. We proved that GASL1 was downregulated in CHF and predicted survival. Moreover, our in vitro experiments provided evidence that GASL1 overexpression may improve CHF by downregulating TGF-β1.

With the efforts made in the treatment and prevention of CHF, the mortality rate of sudden death among CHF patients has dramatically decreased over the past several decades [12]. However, the overall mortality rate in those patients is still high and the cost of clinical treatment of this disease is a heavy burden on public health [13]. Therefore, it will be important to identify CHF patients with high risk of death and develop individualized therapeutic approaches to improve the survival of those patients. GASL1 was downregulated in cancer development [9, 10]. In the present study we showed that GASL1 was downregulated in plasma of CHF patients and low levels of plasma GASL1 were closely correlated with the high mortality rate of CHF patients. Therefore, plasma GASL1 has predictive value for the survival of CHF patients. However, more clinical studies are needed to further confirm our conclusions.

TGF-β signaling is activated during the development of CHF [14]. Activated TGF-β signaling promotes the apoptosis of cardiomyocytes, thereby promoting the development of CHF [15]. In effect, inhibition of TGF-β is considered as a promising therapeutic target for CHF [15, 16]. Previous studies have shown that TGF-β can regulate the expression of lncRNAs [17]. A recent study reported that TGF-β signaling can also be inactivated by an lncRNA which is named GASL1 [10]. In the present study we showed that GASL1 was also an upstream inhibitor of TGF-β1 in AC16 cells. Moreover, the interaction between TGF-β1 and GASL1 participated in the regulation of the apoptosis of AC16 cells.

It is worth noting that TGF-β1 only partially recovered the inhibited apoptosis of AC16 cells by GASL1. Therefore, GASL1 may also interact with other cellular factors to regulate AC16 cell apoptosis.

## Conclusions

In conclusion, GASL1 was downregulated in CHF. GASL1 overexpression may improve CHF by inhibiting cardiomyocyte apoptosis through the inactivation of TGF-β1.

## Data Availability

The datasets generated and/or analyzed during the current study are not publicly available due to research design, but are available from the corresponding author on reasonable request.

## Abbreviations

CHF: chronic heart failure
ELISA: enzyme-linked immunosorbent assay
FBS: fetal bovine serum
lncRNAs: long non-coding RNAs
PI: propidium iodide
